# Prediction of the permeability of antineoplastic agents through nitrile medical gloves by zone classification based on their physicochemical properties

**DOI:** 10.1186/s40780-020-00179-3

**Published:** 2020-11-02

**Authors:** Toyohito Oriyama, Takehito Yamamoto, Katsuhiko Nara, Yohei Kawano, Katsuyoshi Nakajima, Hiroshi Suzuki, Takao Aoyama

**Affiliations:** 1 Tokyo University of Science, Faculty of Pharmaceutical Sciences, 2641 Yamazaki, Noda, Chiba, 278-8510 Japan; 2 Department of Pharmacy, The University of Tokyo Hospital, Faculty of Medicine, The University of Tokyo, 7-3-1 Hongo, Bunkyo-ku, Tokyo, 113-8655 Japan; 3 The Education Center for Clinical Pharmacy, Graduate School of Pharmaceutical Sciences, The University of Tokyo, 7-3-1 Hongo, Bunkyo-ku, Tokyo, 113-0033 Japan

**Keywords:** Antineoplastic agents, Medical gloves, Permeability, Physicochemical properties, Molecular weight, Nitrile, Zone classification

## Abstract

**Background:**

Permeability of antineoplastic agents through medical gloves is an important factor that must be considered for the appropriate selection of gloves. However, predicting the permeability of antineoplastic agents through medical gloves based on their physicochemical properties remains difficult. Thus, this study aimed to elucidate the relationship between the physicochemical properties and permeability of antineoplastic agents through medical gloves. Additionally, we tried to predict the risk of permeation of antineoplastic agents through medical gloves based on physicochemical parameters.

**Methods:**

Ten antineoplastic agents (carboplatin, carmustine, cisplatin, cyclophosphamide, doxorubicin, etoposide, fluorouracil, ifosfamide, oxaliplatin, and paclitaxel) with varying physicochemical properties were investigated, and their permeation rates (PRs) through nitrile medical gloves of varying thicknesses (0.05, 0.07, and 0.1 mm) were measured using a continuous flow in-line cell device. We also determined the apparent permeation clearance (CL_P,app_) values of the antineoplastic agents based on their PRs at 240 min (PR_240_) and assessed the relationship between CL_P,app_ and physicochemical parameters [molecular weight (MW) and logarithm of octanol-water partition coefficient (LogP)].

**Results:**

The CL_P,app_ values of the 10 antineoplastic agents through nitrile medical gloves (0.05 mm thickness) were significantly correlated with their MWs, but not their LogP values (*P* = 0.026 and 0.39, respectively; Spearman’s rank correlation). This finding indicated that the rates of diffusion of the antineoplastic agents in the glove material showed greater effects on CL_P,app_ than the rates of absorption into the glove surfaces within 240 min of exposure. We then classified the 10 antineoplastic agents into 3 zones (Zone A, high LogP/low MW drugs; Zone B, high LogP/high MW drugs; and Zone C, low LogP) and found that Zones A, B, and C corresponded to high (PR_240_ > 10 ng/min/cm^2^), moderate (PR_240_ < 10 ng/min/cm^2^), and low (no detectable permeation) permeation risk, respectively.

**Conclusions:**

The permeation risk of antineoplastic agents through nitrile medical gloves within the actual continuous wearing time in clinical settings could be predicted using MW and LogP values. We believe that the proposed zone classification of antineoplastic agents will be a useful tool for predicting the permeation risk of antineoplastic agents through medical gloves.

## Background

Antineoplastic agents are known for their cytotoxic, mutagenic, teratogenic, and carcinogenic properties [1, 2]. Since healthcare professionals are at a very high risk of exposure to antineoplastic agents during the handling process [3–6], appropriate use of safety cabinet, closed system transfer devices, and personal preventive equipment (PPE) is highly recommended [7–11]. Among the various PPE, medical gloves are the most important because they directly protect the hands, which are at a higher risk of exposure to antineoplastic agents during the handling process than other parts of the body including the face, arms, and trunk. However, several antineoplastic agents, such as cyclophosphamide (CPA), carmustine (BCNU), and fluorouracil (5FU), can reportedly penetrate through medical gloves within 240 min, which is the possible continuous wearing time of medical gloves in clinical settings, thereby exposing the hands of healthcare professionals to these drugs [12]. Therefore, evaluating the permeability of antineoplastic agents through medical gloves to predict the risk of exposure is of critical importance.

Several previous reports have shown that the product characteristics of medical gloves (type of material, thickness, and surface treatment) and the physicochemical properties of antineoplastic agents are the major determining factors of permeability of antineoplastic agents through medical gloves [12, 13]. Regarding the product characteristics of medical gloves, it has been reported that nitrile rubber, compared to other materials such as latex, is less permeable. It has also been shown that the permeability of antineoplastic agents through medical gloves is inversely correlated to the thickness of medical gloves. Additionally, our previous report showed that surface treatment of medical gloves altered the permeability of antineoplastic agents [14]. Regarding the effects of the physicochemical properties of antineoplastic agents on permeability, Wallemacq et al (2006) reported that antineoplastic agents with logarithm of octanol-water partition coefficient (LogP) values > 0.5 tended to exhibit higher permeability through medical gloves than those with LogP values < 0.5. However, Wallemacq et al also observed a large variability in the permeability values of the antineoplastic agents with high LogP values [12]. The possible contribution of molecular weight (MW) to the permeability of antineoplastic agents has been suggested, but the effects of LogP and MW on the permeability of antineoplastic agents through medical gloves remain to be elucidated. Furthermore, previous reports [12, 13] evaluated permeability of antineoplastic agents using permeation rates (PRs) and breakthrough detection time, the time at which PR exceeds the upper limit (10 ng/min/cm^2^) determined based on the American Society of Testing and Materials (ASTM) guidelines [15]. This indicated that these previous reports did not include direct evaluation of basic kinetic parameters, such as permeation clearances, because PR also depended on the concentration of antineoplastic agents used in permeation experiments. Thus, considering these observations, we concluded that the effects of physicochemical properties on the permeability of antineoplastic agents through medical gloves remain incompletely understood, making it difficult to predict the permeability of antineoplastic agents through medical gloves based on their physicochemical properties theoretically.

In this study, we aimed to elucidate the relationship between the physicochemical properties and permeation clearances of antineoplastic agents through nitrile medical gloves, which are widely used for handling antineoplastic agents in clinical settings. For this purpose, we conducted permeation experiments using nitrile medical gloves of varying thicknesses and antineoplastic agents with varying physicochemical properties. Furthermore, we proposed a zone classification of antineoplastic agents based on their MWs and LogP values to enable prediction of the permeability of antineoplastic agents through nitrile medical gloves.

## Methods

### Antineoplastic agents

Among the antineoplastic agents listed in the ASTM protocol D6978–05 [15], the following 10 antineoplastic agents were selected for this study: carboplatin (CBDCA), BCNU, cisplatin (CDDP), CPA, doxorubicin (DXR) hydrochloride, etoposide (ETP), 5FU, ifosfamide (IFM), oxaliplatin (OXA), and paclitaxel (PTX). CBDCA, CDDP, CPA, DXR hydrochloride, ETP, 5FU, IFM, PTX, and OXA were obtained as pharmaceutical products, while BCNU was purchased from Sigma-Aldrich (St. Louis, MO, USA) since BCNU has not been approved as an injection in Japan (Table 1).

**Table 1:** Antineoplastic agents used in this study

Antineoplastic agents	Brand name	C_Test_ (mg/mL)	MW^a)^	LogP^b)^	Ref.^c)^
Carboplatin	PARAPLATIN® INJECTION (Bristol-Myers Squibb K.K.)	10	371.25	−0.46	[16]
Carmustine	Carmusutin (≥ 98%) (Sigma-Aldrich)	3.3	214.06	1.53	[17]
Cisplatin	Randa® Inj. 10 mg/20 mL (Nippon Kayaku Co., Ltd.)	0.5	300.5	−2.19	[17]
Cyclophosphamide monohydrate	Endoxan® 100 mg (Shionogi & Co., Ltd.)	20	261.1^d)^	0.6	[17]
Doxorubicin Hydrochloride	ADRIACIN® Injection 10 (Aspen Japan Co., Ltd)	10	543.5^e)^	1.4	[18]
Etoposide	Lastet® Inj. 100 mg/5 mL (Nippon Kayaku Co., Ltd.)	20	588.6	0.6	[17]
Fluorouracil	5-FU Injection 250 Kyowa (Kyowa Hakko Kirin Co., Ltd.)	50	130.8	−1.0	[19]
Ifosfamide	Ifomide® (Shionogi & Co., Ltd.)	40	261.09	0.86	[20]
Oxaliplatin	ELPLAT® I.V.INFUSION SOLUTION 100 mg (Yakult Honsha Co.,Ltd.)	5	397.29	− 1.6	[21]
Paclitaxel	TAXOL® INJECTION 30 mg (Bristol-Myers Squibb K.K.)	6	853.9	3.7	[22]

^a)^molecular weight, ^b)^logarithm of octanol-water partition coefficient, ^c)^data source of LogP values, ^d)^as anhydride, ^e)^as free base

For use in the experiments, CPA, DXR hydrochloride, and IFM were absorbed in normal saline, while CBDCA, CDDP, ETP, 5FU, OXA, and PTX solutions were used directly. BCNU was absorbed in dehydrated alcohol (33 mg/mL) and further diluted 10-fold (v/v) with water for injection (Otsuka Pharmaceutical Co., Ltd. Tokyo, Japan) to obtain a final concentration of 3.3 mg/mL.

The concentrations of the test solutions used for the permeation experiments (C_Test_, mg/mL) and the MWs and LogP values of the 10 antineoplastic agents are summarized in Table 1 [16–22].

### Medical gloves

Nitrile rubber medical gloves of three different thicknesses (0.05, 0.07, and 0.10 mm) were used in this study. All medical gloves were kindly supplied by Okamoto Industries, Inc. (Tokyo, Japan) after inspection of their thicknesses. The allowance range of thickness was set at specified values ±0.03 mm.

### Permeation experiments

The permeation experiments were performed using an ILC14 continuous flow in-line cell (PermeGear Inc., Hellertown, PA, USA) by the same method described in our previous report [14]. The area of the medical gloves in contact with the antineoplastic agents was 1 cm^2^, and the surface temperature was maintained at 27 °C throughout the experiment following the recommendations of the F739–07 protocol [23]. The receptor solution (purified water) was pumped at a flow rate of 1 mL/min. After adding the antineoplastic agent solution (1 mL) onto the upper side of the medical gloves, the receptor solutions were collected for 0–15, 15–30, 30–60, 60–120, and 120–240 min. Specimens (0.2–0.5 mL aliquots) were then transferred into polypropylene sample tubes and stored at − 80 °C until the assay was performed.

### Analytical procedure

All assays were consigned to Shionogi Pharma CO., Ltd. (Osaka, Japan). CPA, DXR hydrochloride, ETP, 5FU, IFM, and PTX concentrations in the specimens were measured by ultra-performance liquid chromatography-tandem mass spectroscopy. Inductively coupled plasma-mass spectrometry was applied to measure CBDCA, CDDP, and OXA concentrations in the specimens. BCNU concentrations in the specimens were measured by high-performance liquid chromatography-ultraviolet detection.

The limits of quantitation (LOQs, ng/mL) were as follows: CBDCA, 0.95; CDDP, 0.77; CPA, 0.06; DXR 30; ETP, 12; 5FU, 0.3; IFM, 0.03; PTX, 30; OXA, 1.02; and BCNU, 150. Under our experimental conditions, the LOQs could be converted into PRs (ng/min/cm^2^), which were as follows: CBDCA, 0.016; CDDP, 0.013; CPA, 0.001; DXR, 0.5; ETP, 0.2; 5FU, 0.005; IFM, 0.0005; PTX, 0.5; OXA, 0.017; and BCNU, 2.5.

### Evaluation of permeability

Permeation of antineoplastic agents through nitrile medical gloves was evaluated using their PRs, according to the ASTM protocol D6978–05. PR was calculated at each defined time point using the following formula [23]:

1 $$ \mathrm{PR}\ \left(\mathrm{ng}/\min /{\mathrm{cm}}^2\right)=\left(\mathrm{C}\times \mathrm{V}\right)/\mathrm{t}/\mathrm{S} $$

where C, V, t, and S represent the concentration of the antineoplastic agent in the receptor solution (ng/mL), the volume of the collected receptor solution (mL), the exposure time (min), and the area of the glove surface exposed to the antineoplastic agent (1.0 cm^2^), respectively.

The apparent permeation clearance of each antineoplastic agent at 240 min (CL_P,app_) was calculated using the following formula:

2 $$ {\mathrm{C}\mathrm{L}}_{\mathrm{P},\mathrm{app}}\left(\mu \mathrm{L}/\min /{\mathrm{cm}}^2\right)=1{0}^{-3}\times {\mathrm{P}\mathrm{R}}_{240}/{\mathrm{C}}_{\mathrm{Test}} $$

where PR_240_ represents the PR at 240 min from the start of the permeation experiment (ng/min/cm^2^) and C_Test_ is the concentration of the test solution (mg/mL). In this study, CL_P,app_ was used as the surrogate indicator for assessing the intrinsic permeation clearance of antineoplastic agent, which is not affected by C_Test_.

### Statistical analysis

Spearman’s rank correlation was applied to compare the CL_P,app_ values of the antineoplastic agents through nitrile gloves of varying thicknesses and examine the correlation of CL_P,app_ with MW and LogP. In all the statistical analyses, *P* < 0.05 was considered statistically significant.

## Results

### Relationship between permeability of the antineoplastic agents and glove thickness

**Fig. 1:**
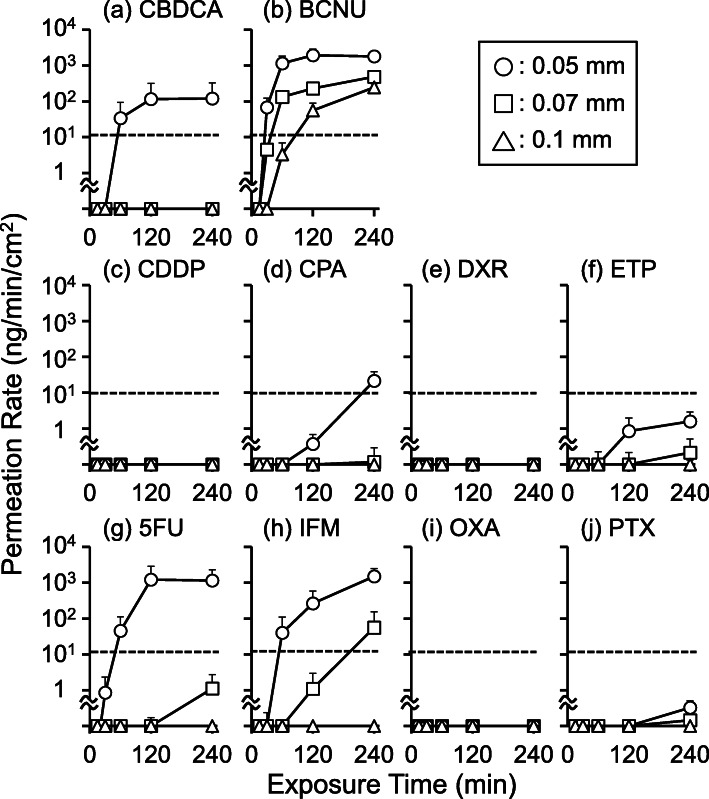
Permeation rates (PRs) of the 10 antineoplastic agents through nitrile gloves of varying thicknesses. The PRs of the 10 antineoplastic agents through nitrile medical gloves are shown. **a**-**j** correspond to carboplatin (CBDCA), carmustine (BCNU), cisplatin (CDDP), cyclophosphamide (CPA), doxorubicin (DXR), etoposide (ETP), fluorouracil (5FU), ifosfamide (IFM), oxaliplatin (OXA), and paclitaxel (PTX), respectively. The symbols and bars indicate mean and standard deviation (SD), respectively (*n* = 3). Open circles, open squares, and open triangles indicate the data obtained using nitrile gloves of 0.05, 0.07, and 0.1 mm thicknesses, respectively. The dotted line indicates the PR upper limit (10 ng/min/cm^2^) determined based on the American Society of Testing and Materials D6978–05 guidelines. Throughout the permeation experiment, CDDP, DXR, and OXA were not detected in the receptor solution

PRs of the 10 antineoplastic agents through nitrile gloves of varying thicknesses are shown in Fig. 1. High PRs (> 10 ng/min/cm^2^) of CBDCA, BCNU, CPA, 5FU, and IFM through nitrile gloves (0.05 mm thickness) were observed within 240 min of exposure. Among these, BCNU, 5FU, and IFM exhibited thickness-dependent decrease in PR. Limited but detectable PRs (< 10 ng/min/cm^2^) of ETP and PTX were observed. Like BCNU, 5FU, and IFM, ETP and PTX also showed thickness-dependent decrease in PR. On the other hand, CDDP, DXR, and OXA exhibited no detectable permeation through nitrile gloves of any thickness at any time point.

**Fig. 2:**
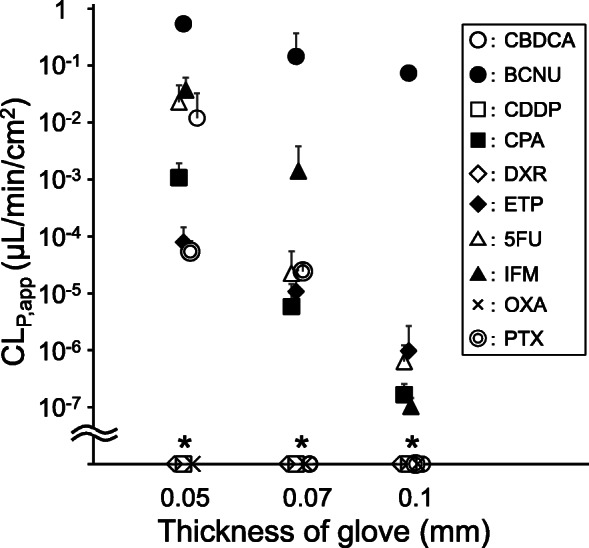
Relationship between apparent permeation clearance (CL_P,app_) values of the antineoplastic agents and glove thickness. The CL_P,app_ values of the 10 antineoplastic agents through medical gloves of three different thicknesses (0.05, 0.07, and 0.1 mm) are shown. The symbols and bars indicate mean and standard deviation (SD), respectively. The symbols on the horizontal axis (indicated with an asterisk) indicate the data of antineoplastic agents that were not detected in the receptor solution throughout the permeation experiments. Statistically significant negative correlation between CL_P,app_ and nitrile glove thickness was observed (Rs = − 0.37, *P* = 0.047, Spearman’s rank correlation coefficient)

As shown in Fig. 2, the CL_P,app_ values of the antineoplastic agents through nitrile gloves were increased with the increase in glove thickness (Rs = − 0.37, *P* = 0.047, Spearman’s rank correlation).

### Relationship between the permeation clearances and physicochemical properties of antineoplastic agents

**Fig. 3:**
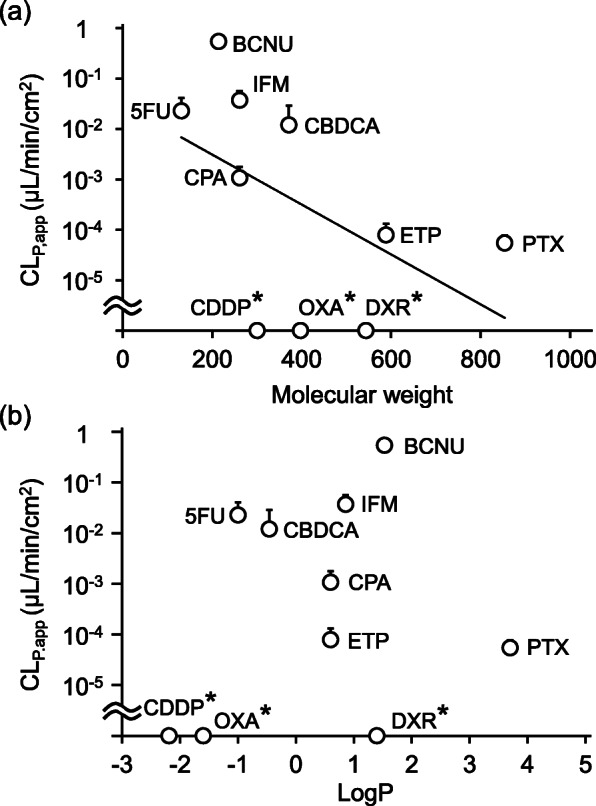
Relationship between the apparent permeation clearance (CL_P,app_) values and physicochemical properties of antineoplastic agents. The relationships between CL_P,app_ of antineoplastic agents through nitrile medical gloves (0.05 mm thickness) and molecular weight (MW) [panel (**a**)] and logarithm of octanol-water partition coefficient (LogP) [panel (**b**)] are shown. Symbols and bars indicate mean and standard deviation (SD), respectively. A statistically significant negative correlation between CL_P,app_ and MW was observed (*P* = 0.026, Spearman’s rank correlation coefficient). In contrast, no statistically significant correlation between CL_P,app_ and LogP was observed (*P* = 0.39, Spearman’s rank correlation coefficient). Since cisplatin, doxorubicin, and oxaliplatin (indicated with an asterisk) were not detected in the receptor solution at 240 min from the start of the permeation experiments, CL_P,app_ values of these antineoplastic agents were calculated as zero

CL_P,app_ values of the 10 antineoplastic agents through nitrile medical gloves (0.05 mm thickness) were plotted against their MWs [Fig. 3(a)] and LogP values [Fig. 3(b)]. As shown in Fig. 3(a), significant negative correlation between CL_P,app_ and MW was observed (*P* = 0.026, Rs = − 0.69, Spearman’s rank correlation). In contrast, no significant correlation between CL_P,app_ and LogP was observed (*P* = 0.39, Rs = 0.31, Spearman’s rank correlation).

## Discussion

The results of this study revealed that MW was the crucial determining factor of permeability of antineoplastic agents through nitrile medical gloves within the possible continuous wearing time of medical gloves in clinical settings (< 240 min). Further, we revealed that the permeability of even the antineoplastic agents with high liposolubility could change based on the MWs. Using these findings, we proposed a zone classification of antineoplastic agents based on their MWs and LogP values to predict permeability through nitrile medical gloves.

**Fig. 4:**
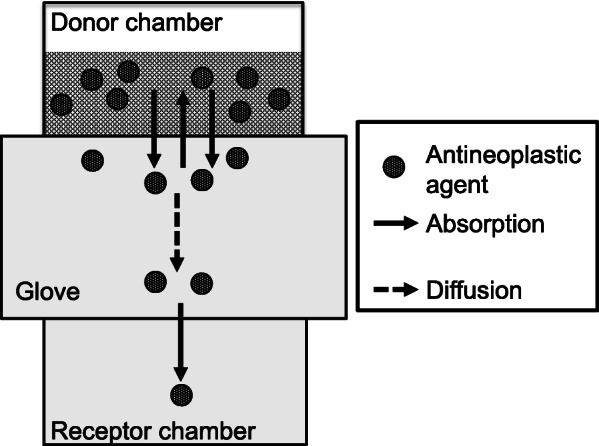
A schematic diagram of the permeation mechanism of antineoplastic agents through nitrile medical gloves. Two mechanisms (absorption and diffusion) were involved in the permeation of antineoplastic agents through medical gloves. After adding the antineoplastic agent solutions onto the upper side of the medical gloves, antineoplastic agents were absorbed into the interface between the glove material and the antineoplastic agent solution. Then, the absorbed antineoplastic agents were diffused in the medical glove material depending on the concentration gradient. Finally, antineoplastic agents were released from the interface between the glove material and receptor solution. Assuming that the diffusion rate constant of antineoplastic agents in the glove material was low, the concentration of the antineoplastic agent in vicinity of the interface between the glove material and receptor solution would increase slowly after the start of the permeation experiments. Thus, permeation rates (PRs) and apparent permeation clearance (CL_P,app_) values also increased in a time-dependent manner until diffusion reached an equilibrium state. This indicats that CL_P,app_ would depended on the diffusion rate constant, which depends on the molecular weight (MW). After diffusion reached an equilibrium state (i.e., after the concentration of the antineoplastic agent in the glove material was uniformized), CL_P,app_ would be no longer time-dependent, but instead absorption rate constant-dependent

As shown in Fig. 1, there was a large variability in the PRs of the 10 antineoplastic agents. Additionally, the slopes of PR against time for five antineoplastic agents (BCNU, ETP, 5FU, IFM, and PTX) with detectable permeation through nitrile gloves of more than one thickness tended to decrease with the increase in glove thickness (Fig. 1). Further, the CL_P,app_ values, which were calculated from the PR_240_ values using Formula-1, were significantly decreased on increasing the medical nitrile glove thickness (Fig. 2). These observations indicated that two mechanisms (absorption and diffusion) were involved in the permeation of antineoplastic agents through medical gloves. To penetrate through medical gloves, antineoplastic agents are absorbed into the medical gloves. Then, the absorbed antineoplastic agents diffuse in the glove material depending on the concentration gradient. Finally, the antineoplastic agents are released into the receptor chamber (Fig. 4). In this study, we set the duration of the permeation experiment to 240 min, which was the possible continuous wearing time of medical gloves in routine clinical settings. However, the PRs of CPA, ETP, IFM, and PTX continued to increase in a time-dependent manner at 240 min (Fig. 1), indicating that the permeation process did not reach a steady state. Because PRs are limited to the lesser of absorption rate and diffusion rate, this observation suggests that either the absorption rate or the diffusion rate limits the PRs of these four antineoplastic agents. Theoretically, antineoplastic agents with high liposolubility (i.e., with high LogP) exhibit rapid absorption into glove material; therefore, the PRs of PTX (LogP = 3.7) would be limited by the diffusion rate. In contrast, the PRs of CPA (LogP = 0.6), IFM (LogP = 0.86), and ETP (LogP = 0.6) would be limited by the absorption rate.

Assuming that antineoplastic agents could diffuse in the glove material by simple diffusion, PRs normalized by C_Test_ values, namely permeation clearances, were expected to be negatively correlated with MWs. As shown in Fig. 3(a), significant negative correlation between the CL_P,app_ values (calculated using Formula-1) through nitrile medical gloves (0.05 mm thickness) and MWs of the 10 antineoplastic agents was observed (*P* = 0.026, Spearman’s rank correlation), indicating that the CL_P,app_ values of the antineoplastic agents were affected by the diffusion rates under our experimental conditions. As shown in Fig. 1(b), though PR of BCNU through gloves of 0.05 mm thickness remained almost constant (approximately 1800 ng/min/cm^2^) 60 min after the start of the permeation experiment, PRs through gloves of 0.07 and 0.1 mm thickness continued to increase in a time-dependent manner even 240 min after the start of the permeation experiment. Since nitrile medical gloves used in this study were the same except for their thicknesses, the observed time lag in PR possibly reflected the difference in the time required for BCNU to diffuse in the glove material. Indeed, Phalen et al (2020) conducted permeation experiments using organic compounds {cyclohexane (LogP = 3.44, MW = 84.16) [17], tert-butanol (LogP = 0.35, MW = 74.12) [24], and cyclohexanol (LogP = 1.23, MW = 100.16) [17]} and nitrile gloves of varying thicknesses and found that the time required to observe detectable permeation of the organic compounds continued to increase on increasing the nitrile glove thickness [25]. Interestingly, the PRs of organic compounds at a steady state were not significantly affected by the nitrile glove thickness. Considering these data, it was indicated that the diffusion rates of antineoplastic agents largely affected the apparent permeation of antineoplastic agents through medical gloves within 4 h of exposure, and thus, the MWs, which affected the diffusion rates, were significant for determining the risk of permeation of antineoplastic agents through medical gloves.

On the other hand, no statistically significant correlation between CL_P,app_ through nitrile medical gloves (0.05 mm thickness) and LogP, which reportedly affected the permeability, was observed [12] (*P* = 0.39, Spearman’s rank correlation) [Fig. 3(b)]. As shown in Fig. 3(b), antineoplastic agents with very low LogP values, including CDDP (LogP = − 2.19, MW = 300.5) and OXA (LogP = − 1.6, MW = 397.29), exhibited no detectable permeation (CL_P,app_ = 0) through nitrile medical gloves, and this observation was consistent with the results of previous reports [12]. This could be attributed to the low absorption rates of CDDP and OXA due to very low LogP values. However, among the 10 antineoplastic agents, 5FU (LogP = − 1, MW = 130.8) exhibited the third highest CL_P,app_ value (0.0228 μL/min) in spite of exhibiting a low LogP value. In contrast, DXR (LogP = 1.4, MW = 543.5) exhibited no detectable permeation through nitrile medical gloves, even though it exhibited the third highest LogP value. Although the underlying mechanisms of these inconsistencies observed with 5FU and DXR were unclear, they could be attributed to the diffusion rates of 5FU and DXR. Considering the very low MW of 5FU (130.8), diffusion rate was expected to be high. Thus, once 5FU was absorbed into the glove material, it could easily and quickly be diffused in the glove material due to its low MW and released into the receptor chamber due to its low solubility. On the other hand, considering the high MW of DXR (543.5), diffusion rate was expected to be low. Although DXR could be easily absorbed into the glove material due to its high LogP value, absorbed DXR could hardly diffuse in the glove material due to its low diffusion rate, and as a result, DXR exhibited no detectable permeation. In other words, the relationship between LogP and CL_P,app_ was much weaker than that between MW and CL_P,app_ under our experimental conditions, making it difficult to detect the statistically significant correlation between LogP and CL_P,app_ in this study.

**Fig. 5:**
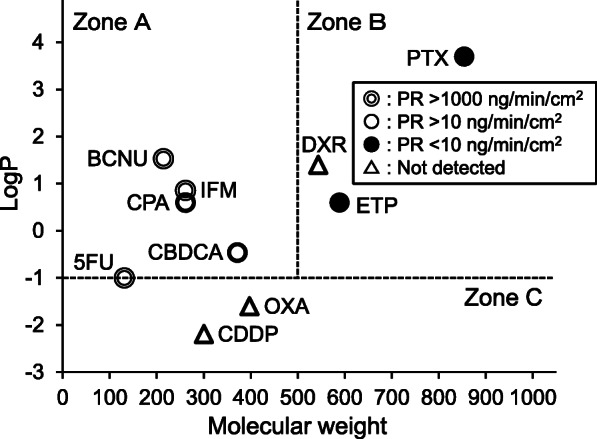
Prediction of the permeation risk of antineoplastic agents through nitrile medical gloves by zone classification. Zone classification of antineoplastic agents based on their molecular weights (MWs) and logarithm of octanol-water partition coefficient (LogP) values proposed to evaluate the risk of permeation through nitrile gloves is shown. The LogP and MW boundaries were set at − 1 and 500 Da, respectively, based on the appearance of the data plot. Each antineoplastic agent was plotted with a different symbol according to their permeation rates at 240 min (PR_240_ values) (see keys in the figure). All “Zone A” (LogP ≥ − 1 and MW ≤500 Da) antineoplastic agents exhibited high PR_240_ values that exceeded the upper limit (10 ng/min/cm^2^) determined according to the American Society of Testing and Materials D6978–05 guidelines. In contrast, the PR_240_ values of antineoplastic agents classified in “Zone B” (LogP ≥ − 1 and MW > 500 Da) were lower than 10 ng/min/cm^2^ (No detectable permeation was observed for doxorubicin). For oxaliplatin and cisplatin, which were classified in “Zone C” (LogP <− 1), no detectable permeation was observed 240 min after the start of the permeation experiments

**Fig. 6:**
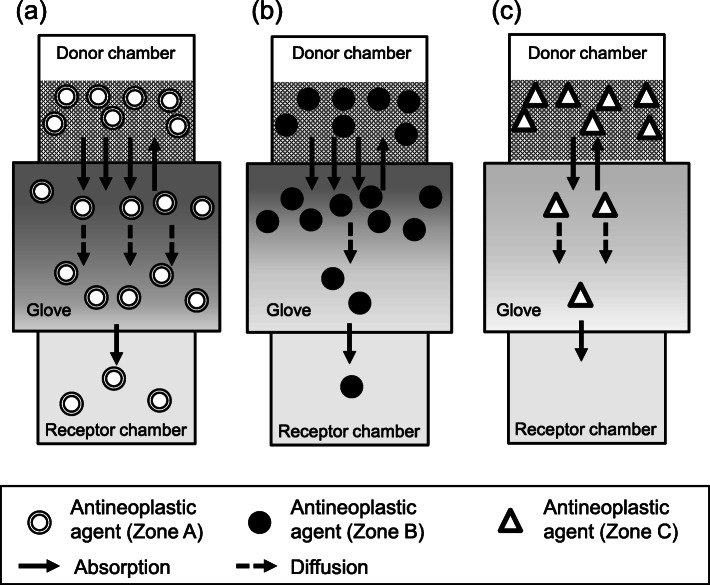
Schematic diagrams of behavior of antineoplastic agents in nitrile medical gloves. The behavior of antineoplastic agents during permeation through nitrile medical gloves is demonstrated. **a**, **b** and **c** correspond to antineoplastic agents classified in Zones A, B, and C, respectively. Since Zone A antineoplastic agents could be easily absorbed into the glove material and easily diffuse to the interface between the glove material and receptor solution, permeation rates at 240 min (PR_240_ values) were expected to be high [panel (**a**)]. In contrast, although Zone B antineoplastic agents could be easily absorbed into the glove material, their rates of diffusion in the glove material were expected to be low due to their high molecular weights (MWs). Thus, PR_240_ values were also expected to be low [panel (**b**)]. Zone C antineoplastic agents could not be easily absorbed into the glove material due to their low logarithm of octanol-water partition coefficient (LogP) values; thus, PR_240_ values were expected to be low irrespective of their MWs [panel (**c**)]

Then, we tried to predict the risk of permeation of the antineoplastic agents through nitrile medical gloves based on their MWs and LogP values. For this purpose, we divided the 10 antineoplastic agents into 3 zones: Zone A, high LogP/low MW drugs (LogP ≥ − 1 and MW ≤500); Zone B, high LogP/high MW drugs (LogP ≥ − 1 and MW > 500); and Zone C, low LogP drugs (LogP <− 1). The LogP and MW boundaries (− 1 and 500, respectively) were set based on the appearance of the data plot. As shown in Fig. 5, Zones A, B, and C clearly corresponded to high [PR_240_ exceeds the upper limit (10 ng/min/cm^2^) determined based on the ASTM guidelines], moderate (PR_240_ < 10 ng/min/cm^2^), and low (no detectable permeation) permeation risk, respectively. The rationale of the zone classification (Fig. 5) is demonstrated in Fig. 6. As shown in Figs. 5 and 6, the Zone A antineoplastic agents demonstrated considerable permeation risk, whereas Zone C antineoplastic agents demonstrated limited permeation risk. On the other hand, Zone B antineoplastic agents demonstrated low permeation risk, but the risk could increase if the exposure time was prolonged. Interestingly, no significant correlation was observed between CL_P,app_ and LogP when the analysis was limited to Zone A (Rs = 0.60, *P* = 0.29, Spearman’s rank correlation) or when all antineoplastic agents were included (Rs = 0.31, *P* = 0.39, Fig. 3(b)); however, Rs values tended to increase when analysis was limited to Zone A. These results suggest that the PRs of antineoplastic agents classified in Zone A (low MW, high LogP) are affected by the absorption rate to some extent and are consistent with the scheme shown in Fig. 6(a). To the best of our knowledge, such zone classification of antineoplastic agents based on their physicochemical properties to predict risk of permeation through medical gloves has not been reported to date.

Although the zone classification helps appropriately predict permeation risk, Fig. 5 shows several exceptions. First, 5FU exhibited high permeability despite it being plotted on the boundary between Zones A and C. This inconsistent observation may be attributed to its low MW. However, it is necessary to conduct permeation experiments using antineoplastic agents with low LogP and Low MW to accurately determine the cutoff values. For this purpose, dacarbazine (MW = 182.18, LogP = − 0.24) and melphalan (MW = 305.02, LogP = − 0.52) are potential candidates. Second, DXR had much lower PR_240_ than ETP and PTX (Fig. 5), despite the MW of DXR (543.5) being lower than that of ETP (588.6) and PTX (853.9). This observation can be explained by assuming that the LogP value of DXR in the test solution was lower than the reported value. For this study, the LogP value of DXR was procured from the interview form [18], in which the LogP value was measured in a buffered solution (pH 7.4), although the pH of the test solution was 5.0–6.0 [18]. Considering the pKa value of DXR (8.22) [18], it is possible that the actual LogP value of the test solution was lower than the literature value, which may explain the smaller PR240 value of DXR than that of PTX and ETP.

Because the zone classification (Fig. 5) was constructed based on PR_240_ data obtained under specific conditions (i.e., using nitrile rubber glove with thickness of 0.05 mm), direct application of this zone classification should be limited to predict the permeation risk of antineoplastic agents under similar conditions. However, the addition of theoretical considerations to zone classification may allow us to estimate the permeation risk of various antineoplastic agents through medical gloves of various materials and thicknesses. For example, the permeation risk of antineoplastic agents through nitrile gloves with different thicknesses can be estimated by considering changes in the LogP and MW boundaries along with the change in glove thickness. Theoretically, the cutoff values of MW and LogP would decrease and increase, respectively, with an increase in glove thickness. Because most nitrile medical gloves are thicker than 0.05 mm, the permeation risk of an antineoplastic agent is estimated to be minimal unless its physicochemical properties are in the upper left part of Zone A. In addition, the PR values of various antineoplastic agents have been measured using various medical gloves in several reports. Therefore, it seems possible to comprehensively estimate the permeation risk of various antineoplastic agents through medical gloves of various materials and thicknesses by re-organizing the PR values obtained from these reports into a zone classification. Although future validation experiments are necessary, the proposed zone classification will be useful in evaluating the permeation risk of new antineoplastic agents through various medical gloves.

This study has several limitations. First, the permeation experiments in this study were discontinued at 240 min, considering the actual time of handling of antineoplastic agents in clinical settings. As shown in Fig. 1, the PRs of several antineoplastic agents (CPA, ETP, IFM, and PTX) were expected to increase further on prolonging the duration of the permeation experiments. However, we assumed that the possible continuous wearing time of medical gloves in clinical settings would not exceed 240 min; thus, we evaluated the factors that affected the risk of permeation of antineoplastic agents through nitrile medical gloves used only until 240 min. Second, the LogP values used in this study were obtained from existing literature, and not actually measured in this study. Since the LogP values are subject to change based on the experimental conditions (pH of solution), the LogP values of antineoplastic agents at pH values similar to those of the test solutions must be measured in future studies. Third, the zone classification shown in Fig. 5 was developed using nitrile medical gloves of 0.05 mm thickness; therefore, it cannot be directly extrapolated to medical gloves made of different materials and/or of different thicknesses. Since the boundary lines for MW and LogP would change depending on the material and/or thickness of the medical gloves, the same experiments using the medical gloves of interest should be conducted as necessary.

## Conclusion

MW was confirmed as the crucial determining factor of permeability of antineoplastic agents through nitrile medical gloves within the actual continuous wearing time in clinical settings (< 240 min). Additionally, permeability of antineoplastic agents with high LogP values (considered highly permeable) could change based on the MW. We believe that the proposed zone classification of antineoplastic agents based on their MW and LogP values will be useful in predicting the risk of permeability of antineoplastic agents through medical gloves.

## Data Availability

Please contact the corresponding author for data requests.

## Abbreviations

ASTM: The American Society of Testing and Materials
BCNU: Carmustine
CBDCA: Carboplatin
CDDP: Cisplatin
C_Test_: Concentration of the antineoplastic agent in the test solution
CL_P,app_: Apparent permeation clearance
CPA: Cyclophosphamide
DXR: Doxorubicin
ETP: Etoposide
5FU: Fluorouracil
IFM: Ifosfamide
LogP: Logarithm of octanol-water partition coefficient
LOQ: Limit of quantitation
MW: Molecular weight
OXA: Oxaliplatin
PPE: Personal preventive equipment
PR: Permeation rate
PR_240_: Permeation rate at 240 min from the start of the permeation experiment
PTX: Paclitaxel
S: Surface area of the glove exposed to an antineoplastic agent.
t: Exposure time
V: Volume of collected receptor solution
